# Myasthenia Gravis and Depression: A Multifaceted Exploration through Omics and Beyond

**DOI:** 10.31083/AP38754

**Published:** 2025-02-28

**Authors:** Zheng Guo, Yulu Zheng, Lois Balmer

**Affiliations:** ^1^Center for Precision Health, Edith Cowan University, 6027 Joondalup, Australia

The interrelationship between Myasthenia Gravis (MG), an autoimmune neuromuscular disorder, and depression, a common psychiatric condition, is a complex phenomenon influenced by various biological, genetic, and environmental factors [1]. ​Advances in genomics, proteomics, and epidemiology provide critical insights into this connection, highlighting the need for integrative approaches that inform therapeutic strategies and improve patient outcomes. By employing techniques such as causal inference, researchers can better discern the direct and indirect factors affecting both conditions, facilitating a more comprehensive understanding of the biological and clinical linkages between MG and depression.

## 1. Genomics and the Genetic Predisposition to Comorbidity

The genetic architecture of MG and depression suggests potential shared pathways that could predispose individuals to both conditions [2]. Genome-wide association studies (GWAS) have identified numerous loci associated with autoimmune diseases, including MG. Key among these are variants in the human leukocyte antigen (HLA) region, which are crucial in immune regulation [3]. The same genetic variants implicated in autoimmune responses may also influence susceptibility to depression, particularly in the context of chronic illness. For example, polymorphisms in genes involved in the serotonergic system, such as SLC6A4, have been associated with an increased risk of depression, and these may interact with immune-related genes to modulate mood disorders in MG patients [4].

## 2. Transcriptomics: Uncovering Gene Expression Changes

Transcriptomic studies provide a window into the gene expression changes that occur in MG patients, especially those with concurrent depression. Analysis of RNA sequencing data from MG patients has revealed differential expression of genes involved in neurotransmission, immune response, and cellular stress, ultimately affecting muscle function [5]. Concurrently, inflammation has been implicated in the pathophysiology of depression, as pro-inflammatory cytokines can impact neurotransmitter metabolism and neuroplasticity [6]. The transcriptional response to such inflammatory processes can illuminate the molecular pathways linking MG and depression, providing a basis for targeted interventions.

## 3. Proteomics: Mapping the Molecular Landscape

Proteomics allows for the identification of protein-level changes that might underlie the link between MG and depression. Previous studies have identified dysregulated proteins that are crucial in neurotransmission and neuromuscular junction integrity, such as acetylcholinesterase and neurofilament light chain proteins [7]. Such alterations in proteomic profiles can influence the development and exacerbation of depressive symptoms in patients with MG. Proteomics can serve as a valuable tool for discovering biomarkers that differentiate between MG-related symptoms and primary depression, enhancing the precision of diagnosis and treatment.

## 4. Metabolomics: Insights into Metabolic Dysregulation

Metabolomic profiles of MG patients often show disruptions in energy metabolism, lipid signaling, and oxidative stress pathways, all of which are also linked to depression [8]. For instance, alterations in tryptophan metabolism, leading to decreased serotonin synthesis, have been observed in both MG and depression [9, 10]. Furthermore, oxidative stress, a hallmark of MG, can exacerbate mitochondrial dysfunction, which is increasingly recognized as a contributing factor in depression [11]. These metabolic disturbances not only affect muscle function but also have systemic effects that could promote depressive symptoms.

## 5. Basic Medicine: The Role of Neuroinflammation

From the perspective of basic medicine, neuroinflammation [12] emerges as a central theme linking MG and depression. The autoimmune nature of MG leads to chronic systemic inflammation [13], which can extend to the central nervous system (CNS). Microglial activation and increased levels of inflammatory cytokines within the CNS can alter neurotransmitter systems, particularly those involving serotonin, dopamine, and glutamate [14]. These changes can disrupt neural circuitry involved in mood regulation, leading to depression. The bidirectional communication between the immune system and the brain—often termed the “neuroimmune axis”—plays a crucial role in this process [15]. Understanding these mechanisms is essential for developing therapeutic strategies that target both the neuromuscular and psychiatric aspects of MG. Such insights can improve the quality of life for MG patients by addressing both their physical and mental health needs.

## 6. Epidemiology: Understanding the Population Impact

Epidemiological studies have consistently shown a higher prevalence of depression among patients with MG compared to the general population [16]. Factors influencing this comorbidity may include the impact of MG on daily functioning, social interactions, and overall mental well-being. Epidemiological research also helps identify risk factors that may predispose MG patients to depression, such as disease duration, severity, and the presence of other comorbid conditions [17]. Additionally, longitudinal studies can track the progression of depression in MG patients, offering insights into the temporal relationship between these conditions and informing the timing of interventions [18].

## 7. Causal Inference: Establishing Directionality and Mechanisms

Employing causal inference techniques enhances the understanding of the relationship between MG and depression by elucidating potential causal pathways. Causal inference methods, such as Mendelian randomization, can be employed to disentangle the directionality of this relationship [19, 20]. By using genetic variants as instrumental variables, researchers can assess whether genetic predisposition to MG increases the risk of depression or if the reverse is true. These methods can also help identify whether the relationship is direct or mediated by other factors, such as chronic inflammation or medication effects [21]. Establishing causality is crucial for developing targeted therapies that address the root causes of depression in MG patients rather than merely treating symptoms.

## 8. Outlook

Integrating insights from genomics, proteomics, and clinical research forms a comprehensive framework to understand the complexities involving MG and depression. The intertwining of these multi-omics realms can potentially revolutionize treatment methods, emphasizing the importance of a multi-faceted approach to health that considers both physical and mental well-being. Further research is needed to uncover the specific mechanisms underlying the comorbidity of MG and depression. Advancements in high-throughput technology and bioinformatics will likely allow researchers to explore these relationships more extensively, pushing the boundaries of current understanding. Additionally, future studies should prioritize patient-centered approaches that include mental health evaluations as part of routine care for those with MG, ensuring a holistic view of patient health.

## 9. Conclusion

The relationship between Myasthenia Gravis and depression is a complex interplay of genetic, molecular, metabolic, and clinical factors. A multi-omics approach, coupled with rigorous epidemiological analysis and causal inference, offers the best opportunity to unravel this complexity and develop comprehensive treatment strategies. Addressing depression in MG requires a holistic approach that considers the biological, psychological, and social dimensions of this comorbidity. By integrating insights from genomics, transcriptomics, proteomics, metabolomics, basic medicine, epidemiology, and causal inference, we can move closer to personalized medicine that addresses both the physical and mental health needs of MG patients, ensuring more effective and patient-centered care.
